# Systematisierung von Methoden partizipativer Forschung

**DOI:** 10.1007/s00103-020-03267-9

**Published:** 2020-12-29

**Authors:** Azize Kasberg, Patrick Müller, Claudia Markert, Gesine Bär

**Affiliations:** Alice Salomon Hochschule Berlin, Alice-Salomon-Platz 5, 12627 Berlin, Deutschland

**Keywords:** Partizipative Forschung, Methodologie, Methoden, Systematisierung, Prozessgestaltung, Participatory research, Methodology, Methods, Classification, Facilitation of research projects

## Abstract

**Hintergrund:**

In der Forschung gewinnen partizipative Ansätze an Bedeutung. Der Einsatz partizipativer Methoden erfolgt meist ohne eine methodologische Auseinandersetzung oder den Bezug auf eine gemeinsame Systematik.

**Ziel der Arbeit:**

Auf der Grundlage eines Scoping-Reviews wird eine Systematisierung partizipativer Methoden vorgeschlagen. Damit wird ein Beitrag zum Diskurs über partizipative Methoden geleistet, die gesundheitliche Chancengleichheit fördern.

**Material und Methoden:**

Nach einer Literaturrecherche wurden 44 Treffer eingeschlossen. Die Suche sowohl nach deutsch- als auch englischsprachiger Literatur basierte auf diversen Datenbanken (PubMed, PsycInfo, SocIndex, Livivo, Cochrane, Fachportal Pädagogik), einer Handsuche und einer Suche nach dem Schneeballprinzip.

**Ergebnisse und Diskussion:**

Die Systematisierung kombiniert phasen- und formatbasierte Logiken. Als zentrale Prinzipien in den Definitionen einer partizipativen Methodologie und von partizipativen Methoden werden die Mehrdimensionalität und Phasenintegration berücksichtigt. Daraus abgeleitet werden die Hauptunterscheidung von prozessgestaltenden und forschenden Methoden. Schwerpunkte zeigen sich bei den Erhebungsmethoden und Reflexionsverfahren. Lücken im deutschsprachigen Raum sind bei Methoden gemeinsamer Entscheidungsfindungen, der Auswertung und Verwendung erkennbar.

**Fazit:**

Die Ergebnisse helfen bei der Einordnung verschiedener Ansätze und ihres Grades an Partizipation. Für Forschung und Lehre wird die Aufmerksamkeit auf das Spektrum forschender und prozessgestaltender partizipativer Methoden gelenkt, die es einzusetzen, zu beschreiben und zu vermitteln gilt.

## Abstract (en)

**Background:**

Participatory research methods are gaining interest. In applying these methods, there is often a lack of consideration for methodological issues. Furthermore, a unifying system for categorizing the various approaches does not exist.

**Objectives:**

Based on a scoping review, a categorization of participatory research methods to promote health equity was undertaken.

**Material and methods:**

Included in the review are 44 publications in German and English retrieved from various databases (PubMed, PsycInfo, SocIndex, Livivo, Cochrane, Fachportal Pädagogik). Additional publications were identified by hand searching and using the snowball method.

**Results:**

The methods are categorized by research phase and type of format used. The principles of multidimensionality and phase integration are central to the proposed system of categorization, resulting in a primary distinction between the methods used to facilitate the participatory research process versus methods used for participatory data collection and analysis. The majority of methods identified are used for data collection or reflexive processes. There are gaps in the German literature regarding methods for cooperative decision making, participatory data analysis, and the application of research results.

**Conclusion:**

The classification system provides an overview of available methods and the degree of participation they promote. The system draws attention to the spectrum of methods available, being useful for both teaching and research purposes.

## Einleitung und Zielsetzung

**Figure 1:**
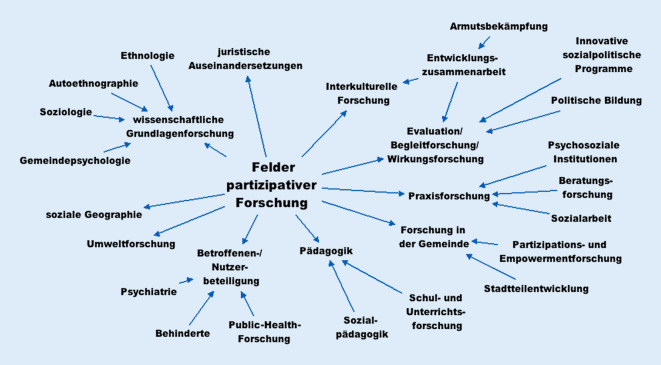

Partizipation ist eine wichtige Aufgabe von Public-Health-Professionellen, um gesundheitliche Chancengleichheit zu fördern. Für ihre Realisierung werden fundierte Kompetenzen und Erfahrungen benötigt. Im Rahmen öffentlich geförderter Forschungsvorhaben der Gesundheitsförderung und Prävention wurden partizipative Methoden modellhaft erprobt und dokumentiert. Gleichzeitig ist es nötig, instrumenteller, symbolischer oder Scheinbeteiligung zu begegnen [1], während die Anwendungsfelder partizipativer Ansätze sehr heterogen sind (Abb. 1).

Der vielfältige Einsatz bei gleichzeitiger methodischer und methodologischer Unschärfe spricht für die Konkretisierung der Methodologie „partizipative Forschung“ und von „partizipativen Methoden“. Die unterschiedlichen Partizipationsverständnisse innerhalb partizipativer Ansätze in der qualitativen Forschung und das damit verbundene Rollenverständnis der mitwirkenden Forschenden (vgl. Kümpers et al. in diesem Themenheft) verweisen zusätzlich auf eine methodische Reflexion partizipativer Methoden.

Ein gemeinsames Verständnis von Partizipation und der Kompetenzaufbau für partizipative Methoden wird in einem Workshopformat im Rahmen des Forschungsprojekts Kompetenzschmiede „Lebenswelten und Gesundheit: partizipative Methoden“ (KLuG) entwickelt. Das Projekt wird durch die BZgA im Auftrag und mit Mitteln der gesetzlichen Krankenkassen nach § 20a SGB V gefördert. Zur Fundierung des Curriculums für kommunale Settings der Gesundheitsförderung wurden Definitionen einer partizipativen Methodologie und von partizipativen Methoden im Unterschied zu partizipativ genutzten Methoden entwickelt. Auf dieser Basis ist ein Literaturreview durchgeführt worden.

Ziel der explorativen Literatur- und Datenbankrecherche war die Erarbeitung eines ersten Systematisierungsvorschlages für die im deutschsprachigen Raum genutzten partizipativen Methoden zur Förderung gesundheitlicher Chancengleichheit.

Der Beitrag soll zum Methodendiskurs innerhalb der partizipativen Gesundheitsforschung (PGF) beitragen, indem er sie methodologisch von anderen partizipativen bzw. Gesundheitsforschungsansätzen abgrenzt.

Diese Überlegungen münden in 3 aufeinander aufbauenden Teilfragen:

- Welche Systematisierungen oder Oberkategorien werden zur Darstellung partizipativer Methoden bereits genutzt?
- Welche Systematisierung lässt sich daraus für den deutschsprachigen Raum ableiten?
- Lassen sich die im deutschsprachigen Raum veröffentlichten Methodensammlungen in den Systematisierungsvorschlag einordnen?

## Konzeptionen partizipativer Gesundheitsforschung (PGF), partizipativer Methodologie und partizipativer Methoden

„Methode“ wird definiert als „auf einem Regelsystem aufbauendes Verfahren zur Erlangung von [wissenschaftlichen] Erkenntnissen oder praktischen Ergebnissen“ [2]. „Die wissenschaftliche Reflexion methodischen Vorgehens bezeichnete man früher als Methodik, heute wird dafür häufig der Metabegriff Methodologie gewählt“ [3].

Ein Grundmerkmal der PGF ist, dass der „Forschungsprozess … zwischen allen Beteiligten partnerschaftlich organisiert und kontinuierlich im Hinblick auf die Machtverhältnisse reflektiert“ wird [4]. Zum definitorischen Kern zählt auch die doppelte Zielsetzung von Erkenntnisgewinn und dem Initiieren sozialen Wandels durch die Forschung [5]. Methodologisch zu berücksichtigen sind die Reflexionsprozesse von Machtverhältnissen und die eigene soziale Positionierung der Forschungsteilnehmenden [6]. Es werden „eine besondere Sensibilität für Gruppendynamiken und die Vermeidung von sozialen Ausschlussformen“ für erforderlich gehalten [6]. Forster und Marent fordern die Integration von 3 Dimensionen im partizipativen Prozess: Entscheidungsteilhabe (Machtdimension), unterschiedliche Wissensformen (Sachdimension) und Phasen (Zeitdimension; [7]), während Cramb und Purcell die Engführung der Phasen „elicitation, analysis, utilisation“ (Erhebung, Auswertung, Verwendung) für den partizipativen Methodeneinsatz fordern [8].

Eine einheitliche Methodologie lässt sich in der vielfältigen Landschaft nicht ausmachen. Begriffe wie „Forschungsstil“ [9] oder „Forschungsstrategie“ [10] versuchen dem auch international vorherrschenden methodologischen Pluralismus gerecht zu werden [11]. Vielfältige Bezüge sind insbesondere zu „qualitativen Methodologien und Methoden“ [9] hervorgehoben worden.

Als Basis valider Partizipationsprozesse und einer strukturierten Vermittlung halten wir es für nötig, die methodologischen Prinzipien partizipativer Ansätze zu einer Definition wie folgt zu verdichten: Partizipative Forschung ist in Abgrenzung zu anderen (Gesundheits‑)Forschungsmethodologien eine Methodologie,

- die sich „in hohem Maße durch Kontextualität und Flexibilität auszeichnet“ [12],
- in der „es gerade darum geht, die Eigensinnigkeit und Eigenwilligkeit der Forschungspartner/innen in dem Forschungsprozess zur Geltung zu bringen“ [6], und
- die „die Initiierung eines offenen Prozesses der zielorientierten Interaktion und der selbstkritischen Reflexion“ in den Vordergrund stellt [6].

Hierfür bedarf es einer Zusammenarbeit auf Augenhöhe. Damit diese gelingt, braucht es die Kombination von analysierenden und prozessgestaltenden Methoden z. B. zur Reflexion geteilter Entscheidungsmacht und von Machtunterschieden [11, 13].

Diesen Prinzipien folgend bedienen sich PGF-Studien einerseits unterschiedlicher Forschungsmethoden der quantitativen und/oder qualitativen Forschung [6, 10, 11] und entwickeln Methoden zur partizipativen Prozessgestaltung im Sinne von gemeinsamem Lernen und Reflektieren, Wissensgenese sowie transformativem Handeln (weiter).

In Anlehnung an Bergold und Thomas [6, 9], Cramb und Purcell [8] sowie von Unger [12] definieren wir partizipative Forschungsmethoden als Methoden,

- die der gemeinsamen Planung und Durchführung von Forschungsprozessen mit Menschen dienen, die vom lebensweltlich situierten Thema und den Ergebnissen des Prozesses betroffen sind, und
- bei denen die 3 Phasen des Methodeneinsatzes (Erhebung, Auswertung, Verwendung) eng verwoben sind und/oder
- die der Selbstreflexion und/oder Reflexion von Machtverhältnissen dienen.

Dagegen zählen zu „partizipativ genutzten Methoden“ neben den Methoden der qualitativen und quantitativen Forschung (bei denen Erhebung und Auswertung ggf. getrennt voneinander erfolgen können) auch Kommunikations‑, Informationsvermittlungs‑, Planungs- und Prozessgestaltungsmethoden.

## Systematisierung partizipativer Methoden

Systematisierungsvorschläge partizipativer Methoden sind wie die erkenntnistheoretischen Fragen in der deutschsprachigen partizipativen Forschung noch wenig diskutiert [14, 15]. Eine der Ausnahmen bildete Wiegering (2015), welche im Rahmen ihrer Diplomarbeit die partizipative Forschungspraxis mit Menschen mit Lernschwierigkeiten formatbasiert systematisierte. Bürgerbeteiligungsverfahren werden beispielsweise anhand einer Mischung von Mitwirkenden, Sozialform, Funktion und Aufwand [16] oder aus Funktion und Phasen im Prozess kategorisiert [17], während in der Praxisforschung phasenbasiert vorgegangen wurde [18]. Eine Quelle der partizipativen Aktionsforschung listet Methoden nach „action“ und „reflection“ auf [19].

## Methodisches Vorgehen des Scoping-Reviews

Nach dem Verfahren eines Scoping-Reviews [20] wurde in nationalen und internationalen wissenschaftlichen Literaturdatenbanken sowie mittels einer Hand- und einer Schneeballsuche recherchiert. Der Einschluss von sogenannter grauer Literatur war notwendig, da partizipative Forschungsprojekte selten in Zeitschriften mit Peer-Review veröffentlicht werden [21].

Mit deutschen und englischen Schlagwörtern wurde nach den fragerelevanten Segmenten national und international mit Bezug zu unterschiedlichen Ansätzen gesucht. Dabei wurde die „Bürgerbeteiligung“ wegen ihrer Verbreitung im deutschsprachigen Raum und der methodischen Überschneidungen ergänzt. Tab. 1 stellt den verwendeten Suchstring der Recherche dar.

**Table 1:** 

Segment	Deutsche Suchbegriffe	Englische Suchbegriffe
Partizipation	Partizipation ODER partizipativ ODER partizipatorisch ODER Mitbestimmung ODER mitbestimmen ODER Kollaboration ODER kollaborativ ODER Mitforschen	Participation OR participatory OR participative OR collaborative OR collaboration
Systematisierung von Methoden	Methode ODER Werkzeug ODER tool ODER Systematisierung ODER Kategorisierung ODER Klassifikation	Method OR tool OR systematization OR systematisation OR categorisation OR categorization OR classification
Ansätze	Bürgerbeteiligung ODER Aktionsforschung ODER Handlungsforschung ODER „Citizen Science“ ODER „Transdisziplinäre Forschung“	CBPR OR PAR OR „action reseach“ OR appraisal OR „citizen participation“ OR „community-based participatory research“ OR „citizen science“ OR „transdisciplinary research“

Die Literaturrecherche erfolgte vom 17.12.2019 bis zum 16.01.2020 in den Datenbanken PubMed, PsycInfo, SocIndex, Livivo, Cochrane und Fachportal Pädagogik. Anschließend wurden mittels Hand- und Schneeballsuche bis zum 01.07.2020 weitere Quellen ergänzt.

In der Metadatenbank PubMed wurde die Suche auf die letzten 10 Jahre beschränkt. In den anderen Datenbanken wurden keine Filter gesetzt. Die Anwendung dieser Suchstrings führte zu 105 Treffern in PubMed, einem Treffer über SocIndex und 14 Treffern über PsycInfo. Die Suche in Livivo, der Cochrane-Datenbank und dem Fachportal Pädagogik führte zu keinen weiteren Treffern. Es wurden 121 Treffer identifiziert, von denen 16 Dubletten waren, sodass 105 Treffer für die Sichtung der Titel, Abstracts und, wenn nötig, der Volltexte verblieben. Diese Artikel wurden von 2 Personen begutachtet.

Als Ein- und Ausschlusskriterien galten die Benennung eines Spektrums partizipativer Methoden und deren Systematisierung und/oder die Nennung möglicher Oberkategorien. Treffer, die sich nicht auf partizipative Methoden im oben beschriebenen Sinne bezogen, wurden ausgeschlossen. Die meisten ausgeschlossenen Treffer verwendeten den Begriff „community-based“ im Sinne von „Gemeinde“ oder „Kommune“ und nicht gemäß von Unger im Sinne von communitybasierter partizipativer Forschung [12] oder entsprechend der Definition von Community der Weltgesundheitsorganisation [22].

Für die Auswertung bzw. Erstellung eines Systematisierungsentwurfes wurden nach Abschluss der Sichtungen 6 Treffer auf Basis der Recherche in (Meta‑)Datenbanken, 37 Treffer durch die Handsuche und 1 Treffer nach dem Schneeballprinzip ermittelt. Aus den 44 eingeschlossenen Treffern wurden potenzielle Kategorien extrahiert und ein erster Entwurf erstellt.

### Entwicklung des Systematisierungsentwurfes

Unter den identifizierten Ansätzen zur Systematisierung der Methoden wurde „Format“ als erster Ansatz zur Systematisierung von partizipativen Forschungsmethoden ausgewählt. Die Sozialform bzw. Art der Interaktion werteten wir als Eigenschaften einer Methode. Da die Methodologie der partizipativen Forschung definitionsgemäß von Flexibilität geprägt ist, müssen partizipative Methoden an die Bedürfnisse der Mitwirkenden anpassbar sein [12]. In welcher Phase eines Forschungsprojekts eine partizipative Methode genutzt wird, hängt in der Regel eher von dem Einsatz im Forschungsprojekt als von der Methode selbst ab. Wie einleitend dargestellt, halten wir auch prozessgestaltende Methoden für die partizipative Forschung relevant, die zusätzlich nach Funktionen im Prozess kategorisiert werden.

Die identifizierten (Ober‑)Kategorien wurden zusammengefasst, hierarchisiert, zu einem Systematisierungsentwurf zusammengefügt und, wenn nötig, durch weitere strukturierende Zwischenkategorien ergänzt. Zur Übersetzung englischer Kategorien wurde, wenn nötig, Literatur zur qualitativen Sozialforschung, sozialen Arbeit oder Psychologie hinzugezogen.

Im letzten Schritt erfolgte die Überprüfung, ob der Entwurf geeignet ist, im deutschsprachigen Raum veröffentlichte Methodenspektren partizipativer Methoden abzubilden. Dafür wurde die Systematisierung exemplarisch auf 3 Methodensammlungen angewendet: auf den Methodenkoffer der partizipativen Qualitätsentwicklung der Deutschen Aidshilfe e. V. (pQ-DAH [23]), auf die Praxishilfe von QuAKTIV [24] sowie auf die Veröffentlichungen der Forschungsprojekte des Forschungsverbunds PartKommPlus (PKP; [25]). Die in diesen Quellen aufgeführten Methoden wurden anhand der jeweiligen Beschreibungen zugeordnet. Der Entwurf wurde kontinuierlich im Forschungsteam diskutiert und angepasst.

## Ergebnisse

### Darstellung der Treffer

Identifizierbare Ansätze, Systematisierungsgrade, -schwerpunkte und die genutzten Kategorien für die 44 eingeschlossenen Treffer werden im Folgenden dargestellt. Ansätze mit direktem Forschungsbezug machen mit 23 Nennungen etwa die Hälfte der Treffer aus, wobei die partizipative Gesundheitsforschung am häufigsten vertreten ist (Tab. 2).

**Table 2:** 

Partizipative Gesundheitsforschung	8
Aktionsforschung	7
Bürgerbeteiligung	6
Communitybasierte partizipative Forschung	5
Kinder- und Jugendbeteiligung	5
Lehre	4
Participatory Rural Appraisal	3
Partizipative Forschung	2
Partizipative Qualitätsentwicklung	2
Praxisforschung	1
Unklar	1

Eine Durchsicht der Dokumente zeigt, dass insgesamt 10 Treffer ungeordnete Sammlungen partizipativer Methoden beinhalten, in denen Methoden zwar einheitlich nach einem Schema, aber unsortiert dargestellt werden, was überwiegend auch auf die PGF-Treffer zutrifft (z. B. [23]). Die meisten Treffer (*n* = 34) bieten lediglich einzelne „Oberkategorien“ für eine mögliche Systematisierung partizipativer Methoden (z. B. „arts-based“, kunstbasiert) [26]). Hingegen scheint der Systematisierungsgrad der sortierten, standardisierten Darstellung partizipativer Methoden in den Ansätzen Bürgerbeteiligung (z. B. [27]) und Lehre (z. B. [28]) größer zu sein als in denen, die Partizipation explizit im Sinne der geteilten Entscheidungsmacht interpretieren.

Zur Kategorisierung sind unterschiedliche Ansätze einer Sortierung zu finden. Die Treffer verdeutlichen, dass schwerpunktmäßig anhand der Art des Formates (z. B. [29]) oder orientiert an Phasen (z. B. [27]) vorgegangen wird (Tab. 3). Systematisierungen, die die Gruppengröße/Sozialform (z. B. [30]) oder Eigenschaften der Mitwirkenden (z. B. [31]) nutzen, kommen deutlich seltener vor. Allerdings werden auch Mischformen präsentiert (z. B. [32]), sodass die Zuordnungen nicht eindeutig und Mehrfachnennungen möglich sind.

**Table 3:** 

Phase	25
Format	25
Sozialform	9
Mitwirkende	4
Undeutlich	34

Die Methodenspektren der eingeschlossenen Treffer sind weiter gefasst als die von uns vorgenommene Definition partizipativer Methoden. Die zur Vermittlung von Informationen genutzten Methoden, welche ggf. partizipativ genutzt werden, ordneten wir wegen ihres Didaktikbezugs der Pädagogik zu. Es konnte keine Systematisierung identifiziert werden, die nach der in diesem Review genutzten Definition der Methodologie partizipativer Forschung und partizipativer Methoden in der Lage gewesen wäre, Kategorien für alle partizipativen Methoden zu bieten. Im Folgenden präsentieren wir daher einen eigenen Vorschlag.

### Systematisierung partizipativer Methoden

**Figure 2:**
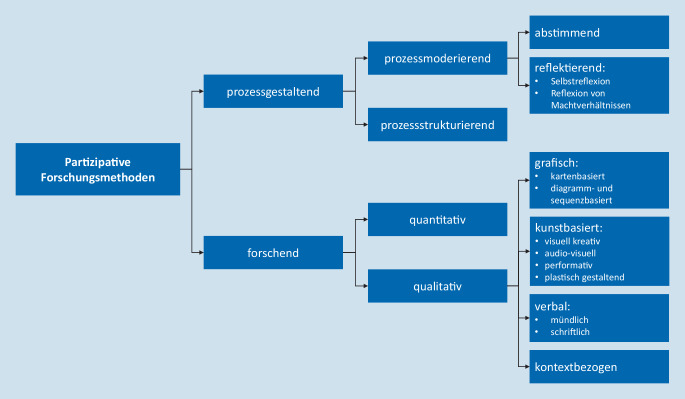

Aus den einzelnen identifizierten Kategorien wurde ein Systematisierungsentwurf für partizipative Methoden im Sinne der hier genutzten Definition erstellt. Abb. 2 zeigt den erarbeiteten Systematisierungsentwurf und Tab. 4 die zugrunde liegende Definition der einzelnen Kategorien und methodischer Beispiele.

**Table 4:** 

**1. Prozessgestaltende partizipative Methoden**	Dienen der partizipativen Gestaltung des Forschungsprozesses
1.1 Prozessmoderierende Methoden	Dienen der Moderation
1.1.1 Abstimmende Methoden [32, 33]^a^	Dienen der gemeinsamen Entscheidungsfindung
	Beispiele: Nominalgruppentechnik [34], Consensus Voting [35], VotAR [36]
1.1.2 Reflektierende Methoden [37, 38]	Dienen der Reflexion und den einwirkenden Machtverhältnissen zur Qualitätssicherung [6]
1.1.2.1 Selbstreflektierende Methoden	Dienen der individuellen Reflexion von Interessen, Leerstellen und Beziehungen [6]
	Beispiel: Forschungstagebücher [39]
1.1.2.2 Machtverhältnisse reflektierende Methoden	Dienen der Analyse von Machtstrukturen und Formen der Beteiligung [6]
	Beispiel: Kreise der Entscheidung [40], Messung der Partizipation in ElfE/ElfE^2^ [41]
1.2 Prozessstrukturierende Methoden [42, 43]	Dienen der Etablierung partizipativer Strukturen im Forschungsprozess
	Beispiele: angeleitete Arbeitsgruppen [44], Nutzerbeiräte [45]
**2. Forschende partizipative Methoden**	Dienen der Gestaltung partizipativer Datenerhebungen und -auswertungen
2.1 Quantitative partizipative Methoden	Nutzen „die statistische Verarbeitung von Messwerten“ zur „Quantifizierung bzw. Messung von Ausschnitten der Beobachtungsrealität“ [46]
	Beispiel: (Peer‑)Befragungen als quantitative Querschnittsstudie [47]
2.2 Qualitative partizipative Methoden	Nutzen „Verbalisierungen (oder anderen nichtnumerischen Symbolisierungen …)“ zur Erfassung von „Erfahrungswirklichkeiten“ [46]
2.2.1 Grafische Methoden/*Visualisation Techniques* [48]	Nutzen eigene Symbole und Sprache der Mitwirkenden zur Visualisierung von Themen und Beziehungen [48]
2.2.1.1 Kartenbasierte Methoden [49]	Nutzen Karten zur Visualisierung räumlich-geografischer Aspekte und/oder abstrakter Merkmale [50]
	Beispiele: Community Mapping [51], Nadelmethode [24] und subjektive Landkarten [24]
2.2.1.2 Diagramm- und sequenzbasierte Methoden/*Diagramming and Sequencing Techniques* [52]	Nutzen grafische Elemente zur Erstellung visueller Darstellungen [53]
	Beispiel: *Relationship Maps, Lifelines, Body Maps, Evaluation Wheel* [54], Kreise der Entscheidung [40], Messung der Partizipation in ElfE/ElfE^2^ [41]
2.2.2 Kunstbasierte Methoden [55]	Nutzen Kunstformen zur Generierung, Interpretation Kommunikation und/oder Darstellung von Wissen [56]
2.2.2.1 Visuell kreative Methoden [57]	Nutzen visuell kreative Medien
	Beispiele: Photovoice [58], Autofotografie [24], Planungswerkstatt: Zeichnen [24]
2.2.2.2 Audiovisuelle Methoden [59]	Nutzen audiovisuelle Medien [60]
	Beispiele: partizipatives Filmprojekt [61]
2.2.2.3 Performative Methoden [9]	Nutzen „improvisierte Darstellungen von Lebens- und Problemsituationen“ [6]
	Beispiele: theaterpädagogische Methoden [62], BeratungsSpiele [63]
2.2.2.4 Plastisch gestaltende Methoden	Nutzen die plastische Gestaltung
	Beispiele: Modellbau [24], Mitmachbautage [24]
2.2.3 Verbale Methoden	Nutzen „Symbole in Form von Wörtern“ [64], einschließlich Gebärdensprache
2.2.3.1 Mündliche Methoden	Nutzen mündliche Formen zur „Generierung und Erfassung verbaler Äußerungen“ [65]
	Beispiele: (partizipative) Fokusgruppe [66], Interviews nach dem Appreciative Inquiry [67], (Peer‑)Interviews [68]
2.2.3.2 Schriftliche Methoden [69]	Nutzen schriftliche Formen zur Generierung und Erfassung verbaler Äußerungen [65]
	Beispiele: (Peer‑)Befragungen als quantitative Querschnittsstudie [47], Forschungstagebücher [39]
2.2.4 Kontextbezogene Methoden	Nutzen Faktoren der materiellen, sozialen und einstellungsbezogenen Umwelt (in Anlehnung an [70])
	Beispiele: teilnehmende Beobachtung [71], Begehung [24], Erkundung und Bewertung [24], Stadtteilspaziergänge [72]

^a^Es wurden keine Beispiele in der Rücküberprüfung identifiziert.

Hierbei ist zu beachten, dass wir davon ausgehen, dass der Systematisierungsentwurf und die beispielhaft genannten partizipativen Methoden nicht vollständig sind. Einige Methoden könnten unterschiedlichen Kategorien der Systematisierung zugeordnet werden. Die vorrangig genutzten Mittel und Medien einer Methode waren entscheidend für die Zuordnung zu einer Kategorie.

Außerdem sagt in der Regel weder die Bezeichnung einer Methode (z. B. Workshop, Fokusgruppe) noch deren Zuordnung im Systematisierungsentwurf etwas über das Maß der geteilten Entscheidungsmacht aus. Wie Bär et al. am Beispiel der Fokusgruppe ausführen, liegt es an der konkreten Nutzung dieser Methode in einem Projekt, ob es sich um eine „klassische“ sozialwissenschaftliche, eine partizipativ genutzte „klassische“ oder eine partizipative Fokusgruppe handelt [73].

Entsprechend dem oben dargestellten methodologischen Hintergrund sind 2 große Methodencluster den Darstellungen zu entnehmen, die sich nach ihren prozessgestaltenden und forschenden Zielsetzungen unterscheiden lassen. Erstere lassen sich wiederum nach prozessmoderierenden und prozessstrukturierenden Formaten einteilen. Der forschende Zweig enthält die Methoden der Datenerhebung und Auswertung. Hierbei werden vorrangig Methoden genannt, die im Phasenmodell partizipativer Forschung in der Datenerhebung „in Zyklen von Aktion und Reflexion“ angesiedelt sind [12].

Die Kategorien der in Tab. 4 dargestellten Systematisierung wurden größtenteils induktiv aus den gesichteten Treffern gewonnen (s. Quellenangaben linke Spalte). Wenn Methoden gefunden wurden, die in keine der identifizierten Kategorien passten (z. B. angeleitete Arbeitsgruppen), wurden diese ergänzt (z. B. prozessstrukturierend). Für die Definition der Kategorien (s. Quellenangaben rechte Spalte) wurde ggf. weitere Fachliteratur genutzt. Die Überprüfung der Systematisierung durch die Anwendung der Kategorien auf die genutzten Methoden von pQ-DAH, QuAKTIV und PKP zeigte, dass sich die partizipativen Methoden gut in den Systematisierungsentwurf einordnen ließen. Wenn keine Beispiele aus der Überprüfung vorhanden waren, wurden sie aus anderen Treffern ergänzt. Bei der Zuordnung zeigte sich, dass die Unterscheidung von partizipativ genutzten Methoden und partizipativen Methoden stark von der Beschreibung des Methodeneinsatzes abhängt. Prozessgestaltende Methoden haben ebenfalls Formate wie verbal oder grafisch. Eine weitere „Hybridität“ zeigen Methoden wie „Photovoice“, die verschiedene forschende Formate in sich vereinen (z. B. visuell kreativ, verbal). Den Zuordnungsregeln entsprechend wurden sie dem vorrangig genutzten Format zugeordnet.

Das recherchierte Methodenspektrum sowie die Überprüfung zeigen vor allem Lücken bei Methoden, die der Abstimmung und gemeinsamen Entscheidungsfindung oder der gemeinsamen Auswertung und Verwendung dienen oder quantitative Methoden nutzen. Hier sehen wir einen großen Weiterentwicklungsbedarf.

## Diskussion

Mit 44 eingeschlossenen Dokumenten liegt eine Basis für die methodische Systematisierung quer zu den partizipativen Ansätzen vor. Der Methodeneinsatz hat sich offenkundig in den letzten 10 Jahren wesentlich weiterentwickelt (vgl. [6]). Dies gilt jedoch nicht gleichermaßen für die Methodendiskussion und die Schärfung der Methodologie. Die fehlenden Systematisierungsbeiträge partizipativer Methoden machen dies deutlich. Auch ansatzübergreifende methodologische Darstellungen sind rar (vgl. [12, 15]).

### Diskussion des methodischen Vorgehens

Methodisch zeigte sich, dass die Recherche in (Meta‑)Datenbanken wenig ergiebig war. Eine Vielzahl der Treffer entsprach nicht dem der Recherche zugrunde liegenden Verständnis von Partizipation (z. B. „Community“ im Sinne von „in einer Gemeinde“ [74] oder „PAR“ nicht als Abkürzung für Participatory Action Research nutzen [75]). Die Schwierigkeiten, die sich zudem aufgrund der hohen Methodenanzahl für eine vollständige Rücküberprüfung ergeben haben, zeigen, dass die Debatte mit diesem Beitrag erst einen Startpunkt markieren kann. Über die von uns geprüften Methodensammlungen hinaus gilt es, den Systematisierungsentwurf empirisch weiter auszugestalten. Es ist zudem davon auszugehen, dass durch eine längere und intensivere Recherche in sozialwissenschaftlichen Journalen (z. B. über Web of Science, SCOPUS) und Handrecherche weitere Quellen und Kategorien hätten identifiziert werden können.

### Diskussion der Ergebnisse

Im Vergleich aller Quellen wird deutlich, dass der Systematisierungsgrad in der Bürgerbeteiligung höher ist als in der partizipativen Forschung. Hier zeigt sich der durch die öffentlichen Aufträge zur politischen Bildung und Stadtentwicklung etablierte und didaktisierte Methodendiskurs (z. B. [27]). Zudem liegt der höhere Systematisierungsgrad wegen des Ursprungs der partizipativen Zielsetzung in der „Demokratietheorie“ nahe [6]. Aber auch hier fällt auf, dass der Diskurs eher auf der Ebene von „Werkzeugkoffern“ verbleibt und methodologische oder erkenntnistheoretische Ausführungen selten sind.

Gerade diese Auseinandersetzungen sind wichtig, um nicht einer „Kanonisierung“ [9] bzw. starren „Manualisierung“ [6] Vorschub zu leisten. Kriterien der Prozessoffenheit, Gegenstandsangemessenheit und Passung zum jeweiligen Forschungsteam müssen daher in der systematischen Beschreibung partizipativer Methoden eine größere Berücksichtigung finden.

Sehr deutlich wird die große Nähe partizipativer Ansätze zu qualitativen Methodologien. Im Vordergrund stehen explorative Verfahren, das Erfassen von Erfahrungswirklichkeiten und die Explikation einbezogener Wissensbestände. Gemeinsame Entwicklungen qualitativer und partizipativer Ansätze wurden in ihrer emanzipatorischen Stoßrichtung unter dem Begriff „liberationist approaches“ [76] markiert. Eine methodologische Auseinandersetzung zum Stellenwert von Partizipation in den jeweiligen Ansätzen qualitativer Forschung wird vor allem für den deutschsprachigen Raum angemahnt. Graßhoff et al. sehen Defizite in diesem spezifischen Methodendiskurs darin, dass die strukturalistisch-rekonstruktiven Ansätze dominieren, das qualitative Paradigma selbst noch in der Etablierung ist und durch die wohlfahrtsstaatlichen Leistungen die professionelle Perspektive häufig überwiegt [77].

In Bezug auf die Kategorienbildung soll aus Platzgründen lediglich auf die intensivere, kategorisierende Fachliteratur zu Reflexionsmethoden verwiesen werden. In einem Überblicksbeitrag nutzen Wihofszky et al. als Einordnungsschema von Reflexionsformaten den Grad der Strukturiertheit und Formalisierung [41]. Dieses Vorgehen war hier nicht anwendbar, da partizipative Methoden per genutzter Definition systematische Verfahren sind. Alexander et al. wiederum explizieren für Reflexion in der Gesundheitsförderung (2020) 3 Formen: „reflexivity in action“, „reflexivity on action“ und „reflexivity underlying action“ [78]. In unsere Systematisierung entspräche „Reflexion von Machtverhältnissen“ dem Typus „reflexivity underlying action“. Bei der Zuordnung einer Methode in die Kategorien „in action“ oder „on action“ stellt sich die Frage nach dem Methodeneinsatz im Prozess. Beide Quellen sind mit der Kategorie der reflektierenden Methoden abgedeckt.

Eine in den Methodenbeschreibungen offenkundige Lücke zeigt sich beim Einsatz von quantitativen Methoden, sodass diese nicht weiter ausdifferenziert werden konnten. Sie werden in Hintergrundtexten zwar regelmäßig erwähnt, werden in der partizipativen kommunalen Gesundheitsförderung im deutschsprachigen Raum allerdings selten als genutzte Methoden dargestellt (Tab. 4). Bemerkenswert ist, dass in der Überprüfung insgesamt nur die quantitative Methode „(Peer‑)Befragung“ identifiziert wurde, die in einem Projekt mit Menschen mit Lernschwierigkeiten genutzt wurde. Im Projekt „Partizipation und Epidemiologie: Von Daten zu Empfehlungen (P&E)“ [79] wurde die „partizipative Epidemiologie“ thematisiert [80]. Eine gezielte Recherche zur Nutzung quantitativer Ansätze in der partizipativen Forschung könnte weitere Erkenntnisse liefern.

## Fazit

Mit diesem Scoping-Review konnte trotz der unzureichenden Literaturlage eine erste Systematisierung partizipativer Methoden für den deutschsprachigen Raum vorgelegt werden. Dieses Vorgehen halten wir für einen geeigneten Weg, die erkenntnistheoretische Fundierung und methodologische Begründung der partizipativen (Gesundheits‑)Forschung voranzutreiben. Als Anstoß für die Diskussion wurden die recherchierten partizipativen Methoden mithilfe eines Kategorienschemas systematisiert dargestellt. Zentrale Prinzipien der partizipativen Methodologie sind die Mehrdimensionalität des partizipativen Prozesses und seine Phasenintegration. Für die Systematisierung ergibt sich damit eine Mischung aus einer phasen- und formatbasierten Betrachtung in Form der präsentierten prozessgestaltenden und forschenden Methoden. Die Rücküberprüfung an Sammlungen partizipativer Methoden im deutschsprachigen Raum konnte die Differenzierung des vorgelegten Entwurfs bestätigen, aber auch einige Entwicklungsbedarfe im Methodenspektrum identifizieren. Es gilt, gezielt partizipative Methoden zu beschreiben, die für Entscheidungsfindungsprozesse und für die gemeinsame Auswertung und Verwendung wesentlich sind, sowie die Einsatzmöglichkeiten quantitativer Methoden in der partizipativen Forschung weiter zu explorieren.

In der Systematisierung liegen große Vorteile in Bezug auf die Qualitätsentwicklung der Methodenausbildung und das Innovationspotenzial zukünftiger Forschung. Zudem könnten Formen instrumenteller, symbolischer oder Scheinbeteiligung durch den Einsatz partizipativer Methoden (im Gegensatz zu den partizipativ genutzten Methoden) leichter verhindert und ggf. identifiziert werden.

Für die Vermittlung partizipativer Methoden in Lehre und Weiterbildung leitet sich eine wichtige Aufgabe ab: Neben partizipativen Methoden zur Erhebung und Auswertung von Wissen gilt es, Grundlagen und partizipative Methoden der Prozessmoderation, (Macht‑)Reflexion sowie zur strukturellen Einbettung der Projekte didaktisch aufzubereiten und curricular zu verankern.
